# Parents’ Needs When Experiencing the Transition to Twin Parenthood

**DOI:** 10.3390/healthcare12121173

**Published:** 2024-06-11

**Authors:** Maria João Freitas, Isabel Sofia Maneta Travanca, Rubén García-Fernández

**Affiliations:** 1 Nursing Research, Innovation and Development Centre of Lisbon (CIDNUR), Nursing School of Lisbon, 1600-190 Lisbon, Portugal; isabelmtravanca@gmail.com (I.S.M.T.); rgarcf@unileon.es (R.G.-F.); 2 Hospital Santa Maria, 1649-028 Lisbon, Portugal; 3 SALBIS Research Group, Faculty of Health Sciences, Department of Nursing and Physiotherapy, Campus de Ponferrada, Universidad de León, 24401 León, Spain

**Keywords:** pregnancy, twin, parenting, transition, nurse, midwife

## Abstract

(1) Background: The transition to twin parenthood is a demanding challenge with a higher risk of maternal and fetal complications during pregnancy and a postpartum period that involves caring for more than one newborn at the same time with similar and simultaneous needs. (2) Aim: To find out about parents’ needs when experiencing the transition to twin parenthood and to describe the intervention of their specialized nursing support network. (3) Methodology: A descriptive exploratory study, based on a proper non-probabilistic sample of 15 nurses and 55 couples who are parents of twins, using two online questionnaires publicized on social networks. (4) Results: The couple’s needs were identified through knowledge of their experiences and difficulties during pregnancy and after the twin birth. Couples’ and nurses’ perceptions differed on the identified needs. The specialized nursing support network focuses its intervention on providing informative guidance on twin pregnancy and postpartum period, health education, group sharing experiences, home visits, planning, and including a family support network in the management of twin care and the creation of a daily routine. (5) Conclusions: There is a need to implement a program focused on the needs of parents of twins, promoting realistic expectations for the birth and parenting of twins, preparing parents, improving their well-being, and creating a specialized nursing support network available to this population.

## 1. Introduction

The incidence of twin pregnancies has increased in recent decades due to delayed maternal age and the use of assisted reproductive methods. This is associated with a higher risk of maternal and fetal complications, as well as the risk of premature birth [1]. The transition to parenthood is a demanding and complex period, characterized by great instability and vulnerability, experienced differently by everyone, requiring the couple to acquire new skills and behaviors, personal and relationship restructuring, identity and life reorganization, and the development of strategies to adapt to a new reality [2]. Twin pregnancy adds greater vulnerability to this period for the couple, as they need to adapt to parenting twins, with the postpartum period being a more demanding challenge involving caring for two or more children with similar and simultaneous needs [1,3].

Twin childbirth can also be a stress factor, as it mostly ends in preterm birth, with the possibility of cesarean section, twins being admitted to Special Care Units, or being separated due to different health conditions, which can negatively interfere with family adaptation [4,5]. Therefore, the birthplace should be chosen in advance, and parents should be allowed to make a prenatal visit to the location [6]. The birth of twins may result in prolonged hospitalizations, requiring couples to manage visits to the twins, maternal recovery and rest, and attention to older siblings, which can make them feel neglectful towards them. During the initial visits, parents should be assisted in adapting to the environment and caring for their twins, to play an active role in their parenthood [6,7]. Nurses should promote bonding and help parents individualize the twins, according to their characteristics, qualities, and unique abilities, advising them to focus on the daily gains of each one [6]. When one twin is discharged and the other remains hospitalized, it implies a new reorganization, as it is necessary to be available to care for both twins. The nurse can advise parents to include extended family in the care of the twin at home and promote the participation of parents in the care of the hospitalized twin [8]. The hospitalization period is an opportunity for parents to receive support and gain confidence in basic care such as feeding, elimination, and bathing the twins. It is important to emphasize that advising and promoting breastfeeding of twins or providing breast milk can help mothers emotionally connect with them [6]. However, feeding twins can be challenging for mothers, and to improve this experience, it is important to prepare for breastfeeding during pregnancy [7], with continuous support from the nurse throughout the process. At home, caring for twins can be exhausting due to different sleep and feeding patterns and the repetitive performance of routines, causing greater sleep deprivation and interruption. After the father’s return to work, the mother sometimes feels lonely and frustrated at not being able to equally care for the twins [9,10,11]. Social support is a strategy to prevent or reduce the level of stress in parents of twins, as it helps develop ways to deal with twins, with support from the maternal grandmother associated with better well-being and mental health and a better marital relationship [12,13,14].

Considering the prevalence of families with twins, it is crucial to assess the specific needs of parents, offering personalized care to prevent and minimize parental stress and its family impact [15]. The occurrence of unplanned multiple pregnancies stands at around 1–2%. However, in modern times, particularly in developed nations, this rate has climbed to 3–4% due to factors such as advancing maternal age and the widespread adoption of assisted reproductive technologies [16]. The specialist nurse plays a vital role in promoting parents’ health, safety, and self-confidence, identifying problems, providing emotional and physical support, offering appropriate strategies, and coordinating an effective support network to strengthen family dynamics [12,15]. The provision of specialized care based on an educational program focused on the needs of parents of twins has shown positive psychosocial results, with improvements in well-being, mood, self-confidence, and feeling more prepared to deal with twin parenthood. Thus, the anticipatory intervention of a specialized nursing network can improve the transition experience to twin parenthood [17].

In the transition to twin parenthood, several challenges arise that can trigger anxiety, parental stress, and postpartum depression. The demanding care of twins can lead to decreased maternal sensitivity, attachment deficits, lower marital satisfaction, increased family conflict, and higher divorce rates [17,18]. Mothers of twins have reported feeling unprepared to deal with childbirth complications and caring for two or more newborns simultaneously. They mention a lack of information, support, and specific guidance during pregnancy and postpartum [15,17], so it would be important for preparation for these couples to begin at the time of the twin pregnancy diagnosis.

Understanding the needs of parents transitioning to twin parenthood and the difficulties they face is essential for the Maternal and Child Health Nurse Specialist to have an anticipatory and more effective intervention, help parents facing the challenges, identify strengths and opportunities, explore resources, and develop coping strategies to experience twin parenthood with greater tranquility and happiness. Therefore, this study aims to understand the needs of parents in the experience of transitioning to twin parenthood and describe the intervention of their specialized nursing support network.

### Research Question

What are the needs of parents in the experience of transitioning to twin parenthood and what is the intervention of their specialized nursing support network?

## 2. Materials and Methods

### 2.1. Study Design and Procedure

Exploratory and descriptive research was carried out using mixed methods with a predominantly qualitative approach, and saturation sampling. Aiming to identify the real needs of couples experiencing the transition to twin parenthood so that nurses can implement interventions promoting a positive transition, a scoping review (SR) was first conducted following the JBI Manual for Evidence Synthesis [19], with a time limit between 2011 and 2022. The objectives were to map the available scientific evidence on the topic and anchor the construction of two distinct data collection instruments, using Google Forms^®^, disseminated on social networks, pages developed for this purpose, and closed groups of parents and nurses. Since direct contact with study participants was not foreseen, informed consent was provided in the questionnaires, where participants declared having read and understood the study objectives and the purpose of the questionnaire, safeguarding compliance with the General Data Protection Regulation, ensuring the security, protection, confidentiality, and anonymity of the provided data, consenting to their use for academic and scientific purposes.

The questionnaires were disseminated through social networks between 2 February and 28 June 2022.

### 2.2. Instrument

#### 2.2.1. First Version of the Instrument

An ‘ad hoc’ data collection instrument was developed for this research, based on the results of the SR, which allowed to list the most common care needs and difficulties experienced by parents of twins, constituting multiple-response questions (quantitative data) in the questionnaires. The first version of the parents’ questionnaire was evaluated by 3 parents who experienced twin parenting, regarding their comprehension, number and difficulty of the questions, time needed to answer, and suggestions for improvement. The respondents needed an average of 23 m to answer the questionnaire, did not present suggestions for improvement, and considered that the questions were easy to understand; however, they considered the questionnaire too long. In the final version, two open questions were withdrawn, which asked for a description of the care received during pregnancy and postpartum.

A preliminary evaluation of the questionnaire for nurses was carried out by 5 nurses. They considered that the questions were easy to understand, requiring an average of 18 min to complete. They suggested including the option “Admission to hospital” in the multiple-choice question “According to your perception and experience, what are the main difficulties of the couple with a twin pregnancy?”, which was integrated into the final version.

#### 2.2.2. The Final Version of the Instrument

The questionnaire for parents was divided into three parts, created by the authors for this research. The first part aimed to characterize the sample in sociodemographic terms (age of each parent, respondent’s gender, education level, number of children, marital status, family composition, and age of the twins). The second part referred to the experience of twin pregnancy (feelings, needs, and care received), with nine questions (one dichotomous, five open-ended, and three multiple-choice), and the third part addressed the postpartum period (feelings, needs, and care received), also with nine questions (five open-ended, three multiple-choice, and one dichotomous). The questionnaire applied to the nurses had a similar structure: the first part consisted of five questions for the socio-demographic and professional characterization of the sample (gender, age, professional qualification, years of total professional experience, and years of experience in maternal and obstetric health); the second part consisted of six questions (three open, two multiple choices and one dichotomous) aimed at characterizing the needs and difficulties of the couples during pregnancy, as well as the care provided by the nurses (feelings, needs and care provided): “According to your perception and experience, what are the main difficulties of the couple with twin pregnancy?”, “If you answered OTHERS in the previous question, say which ones”, “In your opinion, how could the nursing care provided to couples experiencing twin pregnancies be improved?”, “Is the care you provide to the couple with twin pregnancies different to the care you provide to the couple with single pregnancy?” and “If you answered YES, clarify how they are different”. The third part concerned the puerperium/delivery, with a similar structure, with six questions (three open-ended, two multiple-choice, and one dichotomous) characterizing the needs of the couples and the care provided by the nurses: “According to your perception and experience, what are the main difficulties of the couple in twin postpartum?”, “If you answered OTHERS in the previous question, say which ones”, “In your opinion, how could the nursing care provided to couples during the twin postpartum period be improved?”, Is the care you provide to the couple in the postpartum period different to the care you provide to the couple with a single newborn?” and “If you answered YES, clarify how they are different”. In both the second and third parts, the information requested from the nurses was about their experience throughout their professional careers.

The population consisted of parents and nurses from all over Portugal. The non-probabilistic convenience sampling method was used, with inclusion criteria: parents experiencing twin parenthood (twins aged 12 months or less at the time of study participation) and nurses caring for parents/couples experiencing the transition to twin parenthood (with professional experience in maternal and obstetric health of at least 1 year). Given the qualitative nature of the study, the sample size was determined by the principle of data saturation [20,21,22], where data collection was continued until no new themes or additional information emerged. This led us to include 15 nurses and 55 parents/partners as participants.

### 2.3. Data Analysis

For the analysis of quantitative data, descriptive statistical techniques were used (frequency, percentage, mean, standard deviation, maximum, and minimum), using the IBM SPSS Statistics 27^®^ program. For the analysis of qualitative data, content analysis according to Bardin’s guidelines (2020) was used. In this process, all responses were read in two phases, one more floating and the other more exhaustive [23]. Next, the exploration process was carried out through coding operations. The data collected from the questionnaires were coded in R for the parents’ sample and E for the nurses’ sample, followed by the identification number. Subsequently, the content characteristics were analyzed according to the three stages Bardin (2020) recommended: recording unit clipping, numbering and classification, and aggregation into categories [23].

## 3. Results

### 3.1. Characterization of the Sociodemographic Sample of Parents of Twins

In the sample of parents of twins, the average age (M) of respondents was 33.33 years, with the maximum (Max) observed being 44 years the minimum (Min) being 26 years, and the standard deviation (SD) was 4.468 years. The average age of the partner/parent was slightly higher, at 34.84 years (SD = 5.084). Regarding the number of children, the average number was 2.44 children (SD = 1.013; Range 2–5). It was found that all (100%) of the parents who responded to the questionnaire were female. All sociodemographic data can be found in Table 1.

### 3.2. Sociodemographic and Professional Characterization of the NURSES Sample

There was a predominance of females (93.3%) in the sample. The mean age was 40.93 years (SD = 4.045). Regarding academic background, 53.3% held bachelor’s degrees, 26.7% had post-bachelor degrees and specializations in Maternal and Obstetric Health Nursing, and 20% had master’s degrees. Regarding general professional experience as a nurse, the average years of experience was 18.2 years, with the average number of years of experience in maternal and obstetric health being 12.3 years (Min < 1; Max = 32).

### 3.3. Nursing Care Characterization

Many nurses (*n* = 93.3%) assumed they provided different care adapted to the needs of the couple with twins’ pregnancy. In the content analysis, three categories of nursing care emerged: “Health education”, “ Twins Preparation sessions” and “Specialized and multidisciplinary follow-up”. In the category “Health Education”, the expression “Informing couples and providing them with tools/strategies according to the specificities of this pregnancy and the postpartum period” (E2) was highlighted. In the category “ Twins Preparation sessions “, the nurses used expressions such as “Perform sessions to prepare for childbirth, specific to twin pregnancy” (E14), including content such as “informative guidelines related to twin pregnancy” (E8), and “visit to the place of delivery and neonatology unit” (E6), while in the category “Specialized and multidisciplinary follow-up” used expressions such as “A greater specialized follow-up to prepare for the postnatal period” (E13), with the “inclusion of the extended family to help” (E3), and the “Multidisciplinary follow-up with the inclusion of a psychologist in the team” (E15). After the birth of the twins, the nurses, in addition to the categories listed above, identified the care category “Home visit”, as mentioned by E11, “Promote home visits without frequency limit to improve the couple’s self-confidence and autonomy in the care of the newborn and the self-care of the mother/couple”, as well as for “greater support in breastfeeding” (E7) and “help to find a new routine” (E9).

### 3.4. Needs of the Couple during the Transition to Twin Parenthood and Specialized Support Network

To understand the needs of parents experiencing the transition to twin parenthood, it was essential to comprehend how they experienced this transition during pregnancy and after the birth of the twins, the difficulties they felt, from their perspective and that of the nurses, and the specialized support network used.

#### 3.4.1. During Pregnancy

In terms of the experience of transitioning to twin parenthood during pregnancy, participants felt that the diagnosis of twin pregnancy can arouse a variety of emotions, which can be seen in Table 2.

In the content analysis, five categories of experiences emerged: “Process of Acceptance and Adaptation,” “Emotional Roller Coaster”, “Challenging and Tiring”, “Tranquility”, and “Concern.” Regarding the Process of Acceptance and Adaptation, some of the expressions used by the participants were, “After the first months of internalization and in a process of acceptance…” (R2) and “…Period of adaptation to the idea of having 2 more daughters…” (R36). These statements confirm that the communication of the twin pregnancy diagnosis should be conveyed carefully and reflect the need for time to accept and assimilate the twin pregnancy, with differentiated support being useful to couples if desired. In the Emotional Roller Coaster category, expressions such as “An emotional roller coaster” (R3) and “With a mix of joy and expectation, but also fear…” (R53) stand out.

Regarding the Challenging and Tiring category, expressions like “Very tiring due to the appointments, expectations… Due to the unknown…” (R8) and “It was very challenging, always afraid due to various problems that were being identified” (R29) emerged. Regarding the Tranquility category, expressions like “A calm pregnancy without worries” (R11) and “It was a very calm pregnancy, taken lightly, calmly, without major hiccups” (R44) were used. In the Concern category, the expressions used were, “…always worried if they would survive. If the pregnancy would last until the end” (R10) and “With some uncertainty, but with faith that everything would go well” (R24).

The difficulties experienced by the parents during pregnancy and those detected by the nurses can be seen in Table 3.

#### 3.4.2. Specialized Nursing Support Network

As for the specialized nursing support network, it was found that 25.5% of participants reported not receiving any support from the nursing team, while 74.5% reported receiving support, in the form of informative guidance related to twin pregnancy (80.5%), health education moments in the context of the twin situation (31.7%), about 26.8% reported being provided with support in planning future situations, and 9.8% mentioned having adapted childbirth preparation classes for twins.

#### 3.4.3. After the Birth of the Twins

The experiences of parents in the twin postpartum period were grouped into four categories, “Challenging”, “Mixed Emotions”, “Prematurity and Maternal Recovery”, and “Feelings”. In the Challenging category, expressions like “Very challenging… many uncertainties about what we should do…” (R1) and “Adapting to reality was not easy, taking care of 2 babies is challenging” (R7) emerged. Concerning the Prematurity and Maternal Recovery category, some expressions used were “Very intense due to extreme prematurity and low weight. Almost three months of hospitalization in neonatology with several complications” (R8), “The birth of the triplets was by cesarean section, which involved a difficult recovery period for the mother. The father was undoubtedly the greatest support” (R13), and “Difficult recovery from childbirth, breastfeeding, and sleep deprivation” (R48). In the Mixed Emotions category, expressions such as “With a lot of happiness, but also a lot of despair, stress, insecurity” (R9) and “In a mix of emotions and with a lot of work in taking care of the babies” (R24) stood out, while in the Feelings category, expressions were “Very stressful” (R12), “With many fears and insecurities” (R47), and “In a calm and very happy way” (R32).

The difficulties experienced by the parents after the twins’ birth and those detected by the nurses can be seen in Table 4.

It was found that 34.5% did not receive support and assistance from the nursing team in the postpartum period, while the remaining 65.5% did receive support. When asked how this support and assistance was provided, 52.8% mentioned receiving support when the twins were discharged, 50% mentioned that informative guidance related to postpartum care of twins was provided, 41.7% mentioned that nurses created moments of health education in the context of twin situations, 25% stated that they received home visits from nurses during this period, 13.9% mentioned that these professionals provided sharing of experiences in group settings, 13.9% mentioned that support was provided in planning future situations, and 11.1% mentioned being advised by nurses to include extended family in managing care for twins and creating a daily routine.

## 4. Discussion

The participating parents experienced a variety of emotions upon the diagnosis of a twin pregnancy, such as surprise, insecurity, joy, fear, love, admiration, and sadness, as Heinonen (2022) mentions regarding the impact of the diagnosis on the adaptation process to twin pregnancy and transition to parenthood, which can generate emotional reactions such as distancing, surprise, and confusion [24]. The emotions expressed are sometimes contradictory and conflicting, but the literature clarifies the ambivalence that couples feel, as the risks of the pregnancy can trigger feelings of stress, anxiety, and fear while also being positive and hopeful [25].

As the participating couples described, the experience of a twin pregnancy is physically and emotionally challenging, associated with complications in pregnancy and childbirth, namely, premature birth and the birth of low-birth-weight newborns. It requires an additional process of acceptance and adaptation to the concerns and emotions generated by the awareness of the inherent risks of twin pregnancy [24]. It can be turbulent and tiring, increasing the fear and worry of the couples about its progress, as well as anxiety levels and the need for support. Therefore, it is essential to understand the experience lived by these parents, as pregnancy and the transition to parenthood are critical periods that will influence outcomes for babies and families [5,26].

Considering the difficulties that influenced the transition to twin parenthood, listed by parents and nurses during pregnancy, although similar, their expression/observation assumes different measures or weights in the samples under study. Physical discomfort was the difficulty most cited by couples during pregnancy, while nurses placed greater emphasis on difficulties like anxiety and parental stress. Physical discomfort is explained by the fact that in twin pregnancy, there is an increase in circulating hormonal levels, which can exacerbate common pregnancy disorders, and the presence of two or more fetuses in the maternal uterus increases uterine distension, promoting physical discomfort such as lower back pain, pelvic discomfort, and fatigue [7]. Some studies show the greater susceptibility of twin mothers to developing parental stress due to medical, practical, and financial demands during pregnancy and postpartum [6,7,13,14], which may justify the emphasis given by nurses to this parental difficulty. However, the dissonance between the real difficulties of couples, the care needs they trigger, and the needs that nurses prioritize has important repercussions on the quality of care and the satisfaction of parents with the care they receive. It is important, therefore, to reflect on this finding, understand the causes, and find solutions to mitigate this problem.

Following the arrival of twins, twin parenthood entails encountering a spectrum of emotions. While happiness and joy abound, couples also navigate ambivalence and uncertainty regarding their caregiving capabilities, particularly after the father resumes work. The demanding nature of constant care and attention may leave the mother feeling overwhelmed and drained, questioning her ability to attend to both twins equally. However, with adequate family support and preparation for the twin postpartum period, negative emotions can be mitigated, fostering a renewed and optimistic outlook [12,25]. After the birth of twins, couples face social, emotional, practical, and economic challenges that can affect the emotional well-being of the mother, who often stops caring for herself, isolates herself socially, lives in constant concern for the twins, enters a repetitive family routine, and feels guilty and frustrated for not being able to care for the twins equitably [7,12]. Therefore, emotional, and physical support from partners, family members, and healthcare professionals is essential for the mother to develop a relationship with each twin and respond to each one’s needs [5,6,12], which is consistent with the results of the study that highlights the importance of specialized nursing support network and the format in which it is implemented. After the birth of twins, it is important to adopt family routines; however, due to different sleep and feeding patterns and constant care for twins, couples may have difficulty creating these routines [12]. Additionally, the birth of twins may present an increased risk of maternal complications during labor and the postpartum period, so the mother may need more time to recover physically [12]. The health of the twins and fear of the future are heightened concerns for parents when the birth is premature, requiring more support to reduce the stress and concerns of twin parents [25].

The arrival of twins frequently results in extended stays in the neonatology unit, posing challenges to family adjustment. This necessitates careful management of family dynamics, balancing visits to the twins, maternal recovery, and rest, and attending to the needs of older children [5,6].

Given the experiences reported by participants, which are like those described in the literature, it is understood that extreme fatigue/tiredness is the main difficulty felt after the birth of twins, as other studies report [25,27]. Twin parents are subject to experiencing sleep disturbances, fatigue, and exhaustion due to the burden of care and lack of rest. However, in this study, the nurses underestimated this difficulty, emphasizing parental anxiety and stress, as well as difficulties with breastfeeding. The impact of caring for twins can also lead to decreased self-confidence and parental stress, and can extend to family relationships, resulting in decreased maternal sensitivity, attachment deficits, decreased marital satisfaction, increased family conflict, and higher divorce rates [17,28].

In this regard, Carril-Sen et al. (2014) found that parents feel ill-prepared with a lack of information and support during pregnancy, including inadequate prenatal education classes for twin parents, and concluded in their study that mothers who received these interventions from nurses showed positive psychosocial outcomes, showing improvements in well-being, mood and self-confidence, feeling more prepared for twin parenthood and motherhood [17,28]. Furthermore, it is advisable to begin planning, educating about health, and offering support for twin care immediately upon confirming pregnancy, given the increased risk of complications. In this regard, selecting the birth venue beforehand is crucial. Additionally, and as the nurses said, it is important to schedule a prenatal visit to a neonatology unit, as this can help anticipate the environment, dispel misconceptions, and acquaint parents with the babies’ size, equipment, lighting, and common sounds, thereby reducing anxiety [6]. Similarly, to what was identified by parents and nurses, Roose et al. (2018) also mention that interventions related to emotional, practical, and informational support, as well as group sharing, are crucial for controlling the levels of anxiety and stress experienced by these parents [13,29].

The results of this study highlight the need for more support and specific guidance tailored to twinning, including practical advice on breastfeeding and twin sleep that is tailored to the individual needs of each couple. Thus, the implementation of support interventions for parents during the transition to twin parenthood is essential, with a specialized nursing support network being the key element for support, knowledge sharing, and counseling for twin parents [5,24,27]. Establishing a robust support network spearheaded by a specialist nurse enhances the familial experience for parents of twins. Additionally, forming small groups of twin parents fosters dialogue and the exchange of daily challenges, fostering social interaction and support crucial for sustaining emotional well-being [15]. Additionally, home visits have emerged as a relevant intervention, and in this sense, Carrick et al. (2014) recommend two prenatal and daily visits until the 6th day postpartum, followed by intermittent visits until the 10th day, providing better capacity for parents to face the challenges of twin parenthood [17,18,28].

The study presented provides valuable insight into the needs of parents transitioning to twin parenthood. However, it has certain limitations that must be acknowledged. The use of a non-probabilistic sample may limit the ability to generalize the findings to a broader population of twin parents. Additionally, the reliance on online questionnaires could introduce a selection bias, as only those parents with internet access and social media presence could participate. Lastly, the subjective perception of needs by both parents and nurses might not accurately reflect the objective needs or best practices in twin care.

To further our understanding in this area, it would be beneficial to conduct studies with more rigorous methodologies, such as random sampling or longitudinal studies that allow for long-term follow-up of twin families. Investigating the effectiveness of specific interventions provided by the specialized nursing support network and analyzing the cost–benefit relationship of these support programs would be of great value. Moreover, exploring cultural differences in twin parenthood experiences and how interventions can be adapted to be culturally appropriate and sensitive would be an interesting avenue for research.

## 5. Conclusions

During the conducted study, we observed that parents of twins experience a wide range of emotions, some of which are contradictory, throughout the transition process to twin parenthood. During this process, they face fears, uncertainties, and concerns, and they have individual and specific needs that require information and guidance related to the twins’ situation. From the moment the pregnancy is confirmed until the end of the postpartum period, having a specialized and family support network is crucial due to the potential complications and demands associated with the care of twins. Still, in this study, it was found that an important percentage of couples did not receive specialized nursing support in twin and postpartum pregnancies. As for the parents of twins who had this support, it was found that the typology of specialized care they received is consistent with the differentiated and personalized care that the nurses declared to provide them. However, the study also revealed that nurses perceive couples’ difficulties and actual needs differently, devaluing their physical fatigue and placing greater emphasis on the stress to which they are subjected. This finding immediately raises questions about the relevance and adequacy of the nursing interventions they develop, referring to the need for training. In this sense, it is essential to reflect on these findings and establish a comprehensive care model centered on the real needs of these couples, which may include specific health education sessions for parents of twins, providing updated evidence-based information, and offering family support.

In addition, the need for parents of twins to have specialized nursing care to prepare for pregnancy and postpartum is emphasized. Both parents and nurses recognize that greater availability and sensitivity in care is needed, as well as the implementation of a health education program that integrates information and practical guidance, during pregnancy, focusing on the needs of twin parents, promoting realistic expectations for twin birth and parenting, preparing and improving their well-being.

Finally, the creation of a specialized support network, achieved through telephone or online support and home care, proved to be essential for both couples and nurses, and nurses were appointed as facilitators of the transition process to parenthood, since nurses provide care in the couple’s environment according to identified needs and their resources.

## Figures and Tables

**Table 1:** Characterization of the sociodemographic sample of parents of twins.

Academic background	Bachelor’s degree	45.5%
	Master’s degrees	34.5%
	Doctorates	1.8%
Marital Status	Married	52.7%
	Common law marriage	43.6%
	Single	3.6%
Cohabitation	Partner and children	85.5%
	Extended family	9.1%
	Only with children	3.6%
	With parents/in-laws	1.8%

**Table 2:** Emotions presented by participants after the diagnosis of twin pregnancy.

Surprise	69.1%
Insecurity	61.8%
Joy	58.2%
Fear	54.5%
Love	40.0%
Admiration	34.5%
Disgust	10.9%
Hope	7.3%
Confidence	3.6%
Sadness	1.8%
Other	5.5%

**Table 3:** Perceptions of difficulties during pregnancy.

	Difficulties Felt by Parents	Difficulties Detected by Nurses
Physical discomfort	58.2%	53.3%
Parental anxiety	58.2%	93.3%
Fatigue	50.9%	66.7%
Parental stress	18.2%	53.3%
Experiencing ambivalent feelings during twin pregnancy	14.5%	40.0%
Decrease in marital satisfaction	12.7%	13.3%
Pre-existing health problems	9.1%	--
Increased family conflict	5.5%	6.7%
Decreased self-confidence	1.8%	26.7%

**Table 4:** Perceptions of difficulties after twins’ birth.

	Difficulties Felt by Parents	Difficulties Detected by Nurses
Extreme fatigue/tiredness	67.3%	60.0%
Parental anxiety	32.7%	93.3%
Physical discomfort	40.0%	46.7%
Parental stress	30.9%	53.3%
Decreased self-confidence	20.0%	53.3%
Increased family conflict	14.5%	6.7%
Decreased marital satisfaction	10.9%	33.3%
Difficulties in family management	10.9%	66.7%
Financial difficulties	10.9%	40.0%
Difficulty in bonding with twins	5.5%	26.7%
Hospitalization in Neonatology	38.2%	66.7%

## Data Availability

Data are contained within the article.
